# An OMOP Common Data Model pipeline for electrocardiography dataset integration by standardizing signal metadata and heart rate variability features

**DOI:** 10.3389/fbinf.2026.1801714

**Published:** 2026-06-24

**Authors:** Pierluigi Reali, Valentina Tibollo, Gaia Riboni, Pierluigi Plebani, Letizia Tanca, Maria G. Signorini

**Affiliations:** 1 Department of Electronics Information and Bioengineering, Politecnico di Milano, Milano, Italy; 2 Laboratory of Medical Informatics and Artificial Intelligence, Istituti Clinici Scientifici Maugeri IRCCS, Pavia, Italy

**Keywords:** data integration, extraction transform load, findability, health big data, multicentric studies, ontologies, standards

## Abstract

Efficient data sharing is crucial for the secondary use of healthcare data, which represents one of the greatest opportunities to advance pathophysiological knowledge and improve health outcomes. However, data volume and the variety of formats and standards significantly hinder data interoperability, compromising multicentric studies. Enhancing data FAIRness can increase the volume of well-characterized data to train more generalizable, interpretable, and multimodal machine learning models. The extraction and management of high-quality metadata can help institutes describe and represent their data in heterogeneous and unstructured datasets comprising biosignals, bioimages, and omics data. Metadata provides ready-to-use descriptors, enabling researchers to filter the datasets to identify samples of interest for their analyses. The Observational Medical Outcomes Partnership Common Data Model (OMOP CDM) provides a flexible solution for storing metadata from multiple healthcare data types, using unique concepts to ensure effective integration across data sources and semantic interoperability. This work presents an OMOP CDM-based standardization pipeline to extract and enable coherent representation of electrocardiographic (ECG) signal features, including arrhythmias and heart rate variability (HRV) indices, starting from public ECG datasets with different characteristics. We structured the extracted information into OMOP CDM tables and mapped each feature to its closest concept in the standard vocabularies. We found suitable standard concepts for all the ECG annotations and some HRV features. For those features that could not be mapped to standard concepts, we illustrate a workflow to establish special, OMOP-compliant mappings. We discuss the pros and cons of these approaches and propose strategies to improve the management of data standardization pipelines and custom vocabularies in federations. To help researchers test our data integration approach, open-source code covering the entire pipeline is provided. OMOP CDM proved a valid tool for harmonizing ECG metadata. However, its standard vocabularies require extensions to allow describing outputs from advanced signal processing techniques.

## 1 Introduction and preliminary concepts

Health data, metadata, and features, including information derived from biosignals, images, genomic data, and free-text documents, can be stored following diverse approaches. The lack of universally accepted data formats, structures, and vocabularies for the various types of health data leads to considerable syntactic and semantic heterogeneity across data collected by different hospitals, increasing the complexity of data sharing and limiting data reusability in research.

To facilitate the joint analysis of multiple datasets from multicentric studies, in particular ensuring correct interpretation of the results and optimizing the *findability* of relevant data for a specific study within large and heterogeneous repositories, such as data lakes, an effective data integration pipeline is needed. Data integration (or *harmonization*) is the process of combining data from several sources to create a cohesive dataset. This involves reconciling heterogeneous data types, formats, and structures, even from different domains - such as raw data and patient clinical information - into a unified framework that supports interoperability and reuse (Sujansky, 2001; Capuano et al., 2022). The problem of data integration can be analyzed from several perspectives, each related to the different components of the data that require harmonization.

First, harmonization can be performed at the *data content level*, making the data and the extracted features more homogeneous and comparable, thereby reducing the so-called *center effect* that can bias statistical analyses involving data collected from multiple hospitals (Tassi et al., 2024a). The magnitude of the center effect can vary significantly depending on the devices, protocols, and procedures used to collect the data. Consequently, the requirements for data content harmonization and the associated complexity vary across data types. In this sense, biosignals are generally less demanding than other biomedical data, since differences in signal content acquired by clinical-grade devices, as long as standard electrode positioning is followed, are limited to device technical specifications, such as sampling frequency, passband frequency ranges, and signal reference. These differences can often be resolved by applying simple signal-processing techniques (e.g., interpolation to a common sampling rate, re-referencing) to produce final datasets with homogeneous data content. By contrast, bioimages are far more demanding with respect to data content harmonization, as features extracted from Magnetic Resonance Imaging (MRI) data tend to vary across scanners, even within batches of the same manufacturer and model (Tassi et al., 2024b).

In second place, health data carrying the same type of information can be stored using heterogeneous *data formats*. For example, the European Data Format (EDF or EDF+) (Kemp et al., 1992; Kemp and Olivan, 2003), Brain Imaging Data Structure (BIDS) (Gorgolewski et al., 2016), and delimited text formats, such as comma-separated variables (CSV), are especially popular for sharing and analyzing biosignal data. As a result, conversion to a single common format is often required for studies retrieving biosignals from multiple data sources. Bioimages, on the other hand, are less demanding in this regard, as DICOM is the *de facto* standard format for storing, sharing, and analyzing most types of biomedical image data (Mildenberger et al., 2002).

A third fundamental component that generally requires harmonization is *metadata*, which are descriptors that provide information and essential context for the accurate analysis and interpretation of the data (Wilkinson et al., 2016). At the metadata level, harmonization can address.

- The *structure* of metadata: standardized clinical data models, such as the Observational Medical Outcomes Partnership Common Data Model (OMOP CDM) or the Health Level Seven (HL7) Fast Healthcare Interoperability Resources (FHIR) (Bönisch et al., 2022), help to create a coherent representation of metadata gathered from multiple sources.
- The *semantics* of metadata: standardized ontologies of medical terminology (e.g., LOINC or SNOMED) can be utilized to describe procedures, patient information, events, and features relying on a common vocabulary (Liyanage et al., 2015). In practice, this means using an agreed-upon knowledge base to retrieve all the concepts needed to define dataset variables and characteristics.

Generally speaking, the process of integrating two datasets from different hospitals that store the same measurable entities, such as electrocardiography (ECG) recordings, involves several steps. The first step is to identify a *minimum data-* and *metadata-set* of information that the researchers need for their analyses. This step allows retention only of the data and metadata that are actually needed, thereby focusing the efforts required for harmonization. For ECG signals, the *minimum dataset* should specify, for example, which leads must be available and the minimum sampling rate required for all the recordings of a given type. On the other hand, the *minimum metadataset* should include signal-acquisition properties deemed important for evaluating the quality and appropriateness of the signal for specific analyses, such as the sampling rate. Once these minimum sets of data and metadata have been selected, the second step is to identify which variables in the datasets under consideration store these data and metadata. Indeed, it is common for datasets from different sources to use different naming conventions and descriptions for representing the same data and metadata fields. Discrepancies in the definition and description of these fields can be solved by identifying the pairs of variables sharing the same content (either manually or automatically) and associating them with the best-matching concept extracted from a standardized (i.e., pre-selected) set of medical vocabularies. Once the dataset variables containing the data and metadata of interest have been identified, the third step addresses heterogeneity at the data content level through appropriate processing. This includes, for example, converting all quantitative measures in the minimum data and metadata sets to the chosen units of measurement, or, in the case of ECG signals, reconstructing missing ECG leads (Srivastava et al., 2025).

Taken together, these three steps ensure *interoperability* between the data extracted from the two datasets. Scaling this problem to the integration of many datasets made available through a unified big data repository, also *findability* becomes a major issue. Extracting other features from the raw data, in addition to the pre-existing metadata, can improve the characterization of the data stored in the repository and make them more findable, hence reusable. Specifically, these features constitute additional metadata that researchers can query, along with already available basic data properties (e.g., in ECG signals, duration and sampling frequency), to identify data relevant to their studies.

To ensure consistency in the integrated data, comparability across the extracted data descriptors, and traceability of the whole process, the extraction of such additional features, as well as the harmonization steps illustrated above, should be accomplished through a *standardized data integration pipeline*. Substantially, this is a preprocessing pipeline shared across the institutes participating in a research federation. The pipeline performs and documents all the processing steps adopted to transform the raw data - regardless of their original format - into interoperable data that can be analyzed jointly with all the others generated within the federation. The pipeline is *standardized* in that the minimum sets of data and metadata and the main processing steps are defined with a shared approach involving clinicians, clinical researchers, and engineers. Pipeline development, adjustment, and maintenance are performed using collaborative tools, including Git-based code repositories, to foster collaboration across the federation’s institutes, ensure that pipeline changes are always tracked, and enable personalization of the pipeline in separate *branches* in response to specific analysis needs.

**FIGURE 1:**
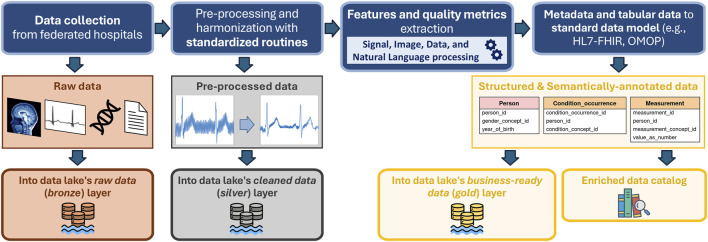
General overview of the standardized data integration pipelines under development in the HBD project.

Within this scope, the Italian Health Big Data (HBD) project (Reali et al., 2024) is developing standardized pipelines specifically designed for the integration and ingestion in a federated data lake of several macrocategories of data, including biosignals, bioimages, omics data, free-text documents, and electronic medical records, collected by the 51 Italian research hospitals involved in the project. Our pipelines address data and metadata harmonization needs in separate conceptual modules, as schematized in Figure 1.

The functions operating within each block can be adjusted based on the needs of the different data types, original data formats, and researchers’ preferences, without drastically affecting downstream components of the pipeline. This is possible because, between any two blocks, a compatibility layer can be inserted to transform the outputs of the previous step and adapt them to the inputs expected by the subsequent block. This pipeline structure facilitates collaboration among developers across different institutes, as it allows keeping the main modules in common, regardless of which institute executes the pipeline. The compatibility layers remain the only components requiring customization for each institute, to shape the original data and potential additional features of interest (i.e., beyond the agreed-upon minimum sets of data and metadata) into the input structure accepted by the subsequent pipeline blocks. Finally, having compatibility layers with dedicated development branches allows contributors to focus processing improvement efforts on the few central modules at the core of the pipelines: new features developed for one of these modules can be easily integrated by repository maintainers after the community discusses and approves them.

The harmonization that standardized data integration pipelines of this kind enable is key to analyzing larger patient cohorts, which are necessary to train accurate and generalizable machine learning and deep learning models. Specifically, it helps satisfy the Findability, Interoperability, and Reusability aspects of the FAIR principles (Wilkinson et al., 2016), making it possible to provide platforms and strategies enabling robust multicentric analyses with data collected from numerous hospitals, which is among the primary outcomes expected from national projects, such as HBD, and international initiatives, such as the European Health Data Space (EHDS).

Following the paradigm illustrated so far, this paper focuses on developing a standardized pipeline to integrate data and metadata derived from ECG signals across different datasets. After preprocessing the original data with established routines, our pipeline extracts features of interest and enables their use as metadata to improve the findability of ECG signals in a federated data lake. We tested the adoption of OMOP CDM to structure these metadata and obtain a semantically rich, unambiguous representation for biosignal information derived from multiple datasets. Specifically, we considered the example of ECG signals retrieved from public datasets and extracted typical ECG annotations, automatically detected arrhythmias, and heart rate variability (HRV) features. Our objective was to develop an adaptable workflow for extracting and mapping these features into a single data model regardless of data origin. For this reason, we relied on several publicly available datasets to simulate part of the heterogeneity observed in ECG recordings acquired across different hospitals.

The significance of this endeavor lies in the fact that only a few works have dealt with the standardization of biosignal-derived information (Choi et al., 2022). In addition, none of these works has comprehensively described the entire process, from feature extraction to OMOP CDM encoding, which we aim to elucidate in this paper.

## 2 Materials and methods

**FIGURE 2:**
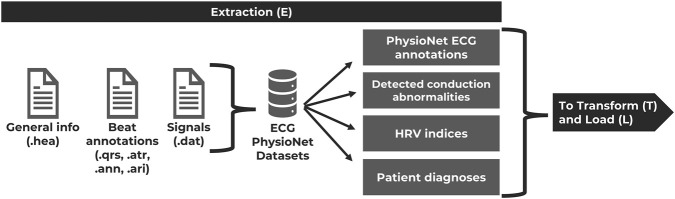
Overview of the ECG pipeline, with a focus on the feature extraction step. Starting from the files composing the original datasets, ECG annotations, automatically-detected conduction abnormalities, HRV features, and patient diagnoses are extracted by applying validated tools.

To reach our goals, a pipeline is needed that (1) imports data from the sources in their original format, (2) processes them with validated approaches to extract features of interest, and (3) structures these features according to a predefined data model, through a dedicated Extract, Transform, Load (ETL) routine. The pipeline consists of functions and scripts developed in MATLAB R2023a (The MathWorks, Inc.) and Python 3.8, leveraging, whenever possible, established and widely tested open-source tools. As strongly advised by the OMOP community, we opted for the relational database management system (RDBMS) PostgreSQL to manage the OMOP CDM database. The current pipeline implementation is made available through a dedicated GitHub repository: https://github.com/hbd-polimi-ws4/ecg2omop-public. While the whole process is summarized in Figure 2, each step will be illustrated in the following sections.

### 2.1 ECG data import and preprocessing

For the development of the pipeline, we considered three PhysioNet (Goldberger et al., 2000) public datasets as ECG data sources, namely the *T-Wave Alternans Challenge Database* (Moody, 2008), the *St Petersburg INCART 12-lead Arrhythmia Database* (Yakushenko, 2008), and the *Lobachevsky University ECG Database* (Kalyakulina et al., 2020; Kalyakulina et al., 2021). The first comprises 100 diagnostic ECG recordings sampled at 500 Hz, most collected with standard 12-lead configuration and lasting approximately 2 min. The second includes 75 30-min 12-lead ECG excerpts from 32 Holter records, each sampled at 257 Hz. The third dataset consists of 200 conventional 12-lead ECG recordings, each collected at 500 Hz and lasting 10 s. Together, the three datasets gather data from healthy controls and patients and include beat-level annotations, such as the locations of P, T, and QRS waves, arrhythmias, and conduction abnormalities, reviewed by experienced physicians.

These datasets were selected to introduce variety to the examined set of ECG signals, in an attempt to partially replicate the heterogeneity found in real-world ECG data. Specifically, part of the considered ECG signals are at least 2 min long, sufficient for robust estimation of several HRV indices, while others resemble standard diagnostic (10-s) or 24-h recordings, broadening the spectrum of analyzed signals to many of the most common categories found in clinical practice. Moreover, most ECG records readily provide beat annotations and other examples of clinical metadata, such as patient sex, age, and primary diagnoses, worth including in the CDM to improve the findability and reusability of demography- or disease-specific ECG signals in health data platforms.

In addition, to test the scalability of the proposed pipeline, we included a fourth large-scale (about 3 GB in size) PhysioNet dataset, the PTB-XL, comprising 21799 12-lead ECG records lasting 10 s each (Wagner et al., 2020; Wagner et al., 2022). Specifically, this dataset was used to evaluate performance metrics on a workstation equipped with an Intel(R) Core(TM) i7-13700KF and 32GB of RAM, running Ubuntu 24.04 on a mechanical hard disk drive.

Every PhysioNet dataset compatible with our pipeline consists of a set of three file categories.

- *Header files*: text files with the extension.hea, containing general information about the ECG trace and the patient, such as the sampling rate, the number and position of electrodes, and the patient’s age, sex, and known clinical diagnoses (when available);
- *Data files*: binary.dat files that collect the ECG signal values sampled for each lead;
- *Annotation files*: variable extension files (e.g., .atr, .ann,. ari, .qrs) reporting on specific waveform locations, arrhythmias, and descriptive comments on clinical evidences found during the study of the trace, detected either visually, by clinicians, or automatically, by the acquisition device. In the latter case, the information provided by annotation files is generally reviewed by clinicians. While header and data files are provided for all datasets, ECG annotations are not always available, and their presence is usually signaled by a file labeled ANNOTATOR, which specifies the reference extension.

The data import and ECG processing procedure starts by scanning the files contained in the dataset directory. A main MATLAB function (MAINextraction) orchestrates and coordinates the activities, calling specific modules and initializing the tables needed to store the data and metadata to be exported at the end of the ETL’s Extraction phase. One of the called modules (COMextraction) extracts, structures, and outputs the various basic signal and patient information contained in the header files. Subsequently, another function (SMPextraction) reads the binary ECG data files and returns the sampled voltage values for each ECG record, along with their timestamps and associated lead labels. In particular, when the ECG trace holds all or some of the typical 12 standard leads adopted for diagnostic ECG (3 bipolar limb leads, 3 augmented unipolar limb leads, and 6 unipolar chest leads), their labels are read from the header file, matched through appropriate regular expressions, and mapped into the corresponding standard lead through a support function (LEADextraction). This is done to address the heterogeneity of ECG channel labels, which are often written with different conventions across datasets (e.g., ECG I, ECG Lead I). The extracted signals are then processed by a fourth function (RRextraction) that determines the duration of the beat-to-beat RR intervals and keeps track of the related R-peak timestamps.

The proper execution of these primary functions relies on auxiliary ones that are called to support specific operations. Further information on those can be found in the pipeline repository linked above.

### 2.2 ECG and HRV feature extraction

For the feature extraction step, we leveraged open-source tools, namely the MATLAB-based *Waveform Database Software Package (WFDB)* (Silva et al., 2021) and the *mhrv* toolbox (Behar et al., 2018). WFDB allows for reading PhysioNet ECG header, data, and annotation files to extract the signals, metadata (e.g., sampling frequency, channel labels, patient information), and standard annotations reported for many PhysioNet datasets (https://archive.physionet.org/physiobank/annotations.shtml), including beat-to-beat annotations like the temporal occurrence of the R peaks, which we employed to derive the RR interval (RRI) time series. The *mhrv* toolbox, instead, enables the identification of R peaks in ECG signals where these are not readily available in the annotation files. Furthermore, it was used to compute clinically established time-domain, frequency-domain, and non-linear HRV indices (Shaffer and Ginsberg, 2017; Sassi et al., 2015). Specifically.

- RRI average (AVRR), standard deviation (SDRR), and root mean square of successive differences (RMSSD);
- Very-low (VLF, 0.003–0.04 Hz), low (LF, 0.04–0.15 Hz), high-frequency (HF, 0.15–0.4 Hz) powers in absolute and normalized units, calculated using the approach based on Fast Fourier Transform (FFT), and the ratio between LF and HF powers (LF/HF);
- SD1 and SD2 descriptors from Poincaré plots, short $\left(\alpha_{1}\right)$ and long-term $\left(\alpha_{2}\right)$ scaling exponents of the detrended fluctuation analysis (DFA), and sample entropy (SampEn). DFA and SampEn were assessed using options widely employed in the literature, namely scales (*n* parameter) from 4 to 15 for DFA $\alpha_{1}$ and from 16 to 64 for DFA $\alpha_{2}$ (Peng et al., 1995); m=2 and r=0.2 were selected for SampEn (Sassi et al., 2015).

In the proposed pipeline, HRV indices are calculated using the same methods on all signals to ensure the extracted features are harmonized across datasets and return consistent results when researchers search for ECG signals with specific characteristics. In particular, RRIs are extracted using the algorithm implemented in the gqrs function of the WFDB toolbox (Moody et al., 2022). Since the processed ECG datasets can include patients suffering from diverse diseases, and it is hard to define universal thresholds suitable for all of them, correction of the extracted RRI based on ectopic beat detection is not performed by default to avoid misrepresenting HRV dynamics under certain conditions (Quintana et al., 2016; Zhao et al., 2021). However, the settings used for ectopic beat detection by the *mhrv* toolbox are made easily accessible via the pipeline’s function performing HRV feature extraction (MTRextraction); thus, they can be properly adjusted when processing datasets comprising homogeneous patient groups.

We opted for the above tools because both are natively compatible with PhysioNet dataset formats, and *mhrv* is a widely tested, MATLAB-native tool for extracting HRV indices, thereby allowing us to focus on developing the ETL procedure and defining a suitable data model to store the ECG data and metadata of interest. Moreover, relying on open-source software enables customization of existing tools by forking the original projects, when necessary, to adapt them to the pipeline, and allows tracking of code versions through their unique commit identifiers. Including precise software version information in the CDM can be essential, especially in certain pipelines, for documenting the characteristics of the preprocessing and feature extraction steps applied.

For the automatic detection of relevant conduction abnormalities, we employed the deep neural network (DNN) developed by Ribeiro and colleagues (Ribeiro et al., 2020), which recognizes six types of them, namely 1st degree atrioventricular block (1dAVb), right and left bundle branch blocks (RBBB, LBBB), sinus bradycardia (SB), atrial fibrillation (AF), and sinus tachycardia (ST). Specifically, this DNN was selected based on the promising accuracy levels it achieved on unseen data (F1-scores between 0.870 and 1.000, depending on the classified abnormality) (Ribeiro et al., 2020). Following the authors’ recommendations (Ribeiro et al., 2020), the extracted ECG traces were resampled (i.e., downsampled or interpolated depending on the original sampling rate) to 400 Hz and zero-padded to 4096 samples, if needed, to make them comparable with the training data used for the original DNN. For ECG signals shorter than 8 s or not featuring the requested 12 standard leads, the DNN is not applied to avoid producing unreliable results. Conversely, if the ECG signal exceeds 10 s, it is divided into 10-s segments, and each one is processed to estimate the scores for every abnormality detected by the DNN. Thus, such scores are compared with the thresholds reported by the authors as maximizing the F1-score on their validation set (1dAVb: 0.124, RBBB: 0.070, LBBB: 0.050, SB: 0.278, AF: 0.390, ST: 0.174). If a score exceeds the threshold in at least one segment, the corresponding conduction abnormality is added to the list of detected ones within that specific ECG trace. This way, we annotate all abnormalities detected in each ECG signal and provide interested researchers with metadata reporting on key patient conditions, which is especially valuable for patients for whom more robust diagnostic information, such as clinically confirmed diagnoses, is unavailable. To implement the illustrated approach, we used publicly available code from the DNN developers and complemented it with a Python function that performs predictions on the ECG signal segments passed by our main MATLAB program. We performed this customization by forking the original Git repository in accordance with the principle outlined earlier.

It should be noted that, thanks to the flexible structure of our modular ECG pipeline, users can replace any selected tool with other open-source packages or custom functions that better fit their data. For example, the popular *Neurokit2* Python package (Makowski et al., 2021) could be used as an alternative to the *mhrv* toolbox for HRV feature extraction, particularly for noisy ECG signals, by leveraging MATLAB’s ability to interact directly with Python libraries. Indeed, high noise levels in the processed ECG signals (e.g., motion artifacts during 24-h or exercise stress test recordings) may impair the detection of both conduction abnormalities and R peaks, resulting in poor-quality RRI time series and, in turn, extracted HRV features. Similarly, a consistent presence of ectopic beats can significantly influence HRV estimates or serve as a marker of specific pathological conditions (Quintana et al., 2016). These factors may require adopting preprocessing strategies or detection algorithms different from those considered in the proposed pipeline, or tuning their settings to the specific patient groups being processed. Thus, researchers are encouraged to screen their data for these potential issues and adapt the pipeline’s processing as necessary to ensure accurate application of the Extraction step to their datasets.

The extraction of ECG standard annotations, calculable HRV indices, and automatically detected conduction abnormalities is performed by dedicated functions in the pipeline (ANNextraction, MTRextraction, and DGNextraction, respectively). Following an approach similar to that used for automated ECG diagnosis detection, annotation files are parsed through dedicated WFDB functions to identify the 16 standard pathological annotations typically recognized in PhysioNet datasets (Table 1). If one of these appears at least one time in an ECG signal, the corresponding abnormality is added to the list featured by that specific ECG signal. HRV features are calculated only when the ECG trace duration permits, in accordance with current guidelines on short- and ultra-short-term HRV (Shaffer and Ginsberg, 2017; Castaldo et al., 2019); otherwise, the dedicated function returns null values when robust estimates are not possible. Specifically, AVRR is always returned, regardless of ECG duration; SDRR is estimated for ECG traces lasting at least 30 s; RMSSD, SD1, and SD2 are calculated only for signals equal to or longer than 60 s; LF and HF powers are returned for ECG traces of at least 120 s, whereas VLF power is computed only for 300-s or longer signals; finally, complex non-linear features (SampEn, DFA $\alpha_{1}$ , and $\alpha_{2}$ ) are estimated for signals equal to or longer than 180 s.

**TABLE 1:** Mapped concepts of the pathological annotations evaluated in PhysioNet standard ECG annotations.

Source term	Map strategy	Concept ID	Concept name	Domain
Atrial premature beat	Standard	4115173	Atrial premature complex	Condition
Aberrated atrial premature beat	Standard	4115173	Atrial premature complex	Condition
Ventricular escape beat	Standard	4327066	Ventricular escape beat	Condition
Supraventricular escape beat (atrial or nodal)	Standard	4327066	Ventricular escape beat	Condition
Fusion of ventricular and normal beat	Standard	4092012	Fusion beat	Condition
Fusion of paced and normal beat	Standard	4092012	Fusion beat	Condition
Premature ventricular contraction	Standard	4089462	Ventricular premature complex	Condition
R-on-T premature ventricular contraction	Standard	4089462	Ventricular premature complex	Condition
Bundle branch block beat (unspecified)	Standard	313791	Bundle branch block	Condition
Right bundle branch block beat	Standard	314059	Right bundle branch block	Condition
Left bundle branch block beat	Standard	316998	Left bundle branch block	Condition
Supraventricular premature or ectopic beat (atrial or nodal)	Standard	441872	Supraventricular premature beats	Condition
Atrial escape beat	Standard	4088986	Atrial escape complex	Condition
Nodal (junctional) escape beat	Standard	4166380	Junctional escape beats	Condition
Nodal (junctional) premature beat	Standard	4218739	Junctional premature beats	Condition
Paced beat	Standard	4304202	Rhythm from artificial pacing	Condition

All source terms belonging to PhysioNet ECG annotations could be mapped relying solely on standard concepts available from OMOP vocabularies.

In total, the 16 types of pathological ECG standard annotations found in PhysioNet datasets (Table 1), 15 established HRV features (Table 2), and 6 conduction abnormalities automatically detected by the selected DNN (Table 3) were extracted from the ECG data and prepared for insertion in the CDM. In addition, 7 types of clinical patient diagnoses (Table 4) that were available in some of the processed header files and were retrieved during preprocessing (through the COMextraction function), were added to the set of extracted features and included in the CDM.

**TABLE 2:** Mapped concepts of the extracted HRV indices and ECG signal characteristics.

Source term	Map strategy	Concept ID	Concept name	Domain
AVRR	Standard	3006307	R-R interval (mean value during study) by EKG	Measurement
SDRR	Standard	21491502	R-R interval.standard deviation (heart rate variability)	Measurement
RMSSD	Custom standard	2000000001	Root mean squared successive differences	Measurement
HF power	Custom standard	2000000002	HRV power high frequency (HF)	Measurement
HF power normalized	Custom standard	2000000003	HRV power high frequency (HF) normalized	Measurement
LF power	Custom standard	2000000004	HRV power low frequency (LF)	Measurement
LF power normalized	Custom standard	2000000005	HRV power low frequency (LF) normalized	Measurement
LF/HF	Custom standard	2000000006	Ratio of HRV low and high frequency powers	Measurement
VLF power	Custom standard	2000000007	HRV power very low frequency (VLF)	Measurement
VLF power normalized	Custom standard	2000000008	HRV power very low frequency (VLF) normalized	Measurement
SD1	Custom standard	2000000009	Poincaré plot SD1	Measurement
SD2	Custom standard	2000000010	Poincaré plot SD2	Measurement
DFA $\alpha_{1}$	Custom standard	2000000011	Detrended fluctuation analysis alpha1	Measurement
DFA $\alpha_{2}$	Custom standard	2000000012	Detrended fluctuation analysis alpha2	Measurement
SampEn	Custom standard	2000000013	Sample entropy	Measurement
ECG duration	Standard	3004182	Recording duration by EKG	Measurement
ECG sampling rate	Standard plus Non-standard	4117495 37533243	Sampling – action Digital sampling rate	Observation Observation

Most HRV indices source terms required mapping through custom vocabularies, due to the lack of semantically-close concepts in OMOP vocabularies. The *Domain* column clarifies which features were mapped to the measurement or observation tables, based on the OMOP domain each concept belongs to.

**TABLE 3:** Mapped concepts of the conduction abnormalities detected by the selected DNN.

Source term	Map strategy	Concept ID	Concept name	Domain
1dAVb	Standard	314379	First degree atrioventricular block	Condition
AF	Standard	313217	Atrial fibrillation	Condition
LBBB	Standard	316998	Left bundle branch block	Condition
RBBB	Standard	314059	Right bundle branch block	Condition
SB	Standard	4171683	Sinus bradycardia	Condition
ST	Standard	4007310	Sinus tachycardia	Condition

All source terms belonging to the automatically detected conduction abnormalities could be mapped relying solely on standard concepts available from OMOP vocabularies.

**TABLE 4:** Mapped concepts of the patient diagnoses confirmed by clinicians in the considered PhysioNet datasets.

Source term	Map strategy	Concept ID	Concept name	Domain
Acute MI	Standard	312327	Acute myocardial infarction	Condition
Coronary artery disease	Standard	4168972	Obliterative coronary artery disease	Condition
Earlier MI	Standard	4329847	Myocardial infarction	Condition
Sinus node dysfunction	Standard	317302	Sinus node dysfunction	Condition
Transient ischemic attack	Standard	46272244	Transient ischemic attack due to embolism	Condition
Arterial hypertension	Standard	316866	Hypertensive disorder	Condition
Left ventricular hypertrophy	Standard	4184746	Left ventricular hypertrophy	Condition

All source terms related to confirmed patient diagnoses could be mapped relying solely on standard concepts available from OMOP vocabularies.

### 2.3 Design of OMOP CDM tables and transform phase

The OMOP CDM is an open and flexible tool for standardizing the structure and representation of data from observational studies (Overhage et al., 2012). Data engineers approaching this standard can count on a set of open-source applications maintained by the Observational Health Data Sciences and Informatics (OHDSI) community, making it particularly suited for prototyping and experimentation, a significant advantage for research platforms enabling the analysis of multiple data types.

The key components of OMOP CDM come down to a standardized set of *tables* and interconnected *vocabularies* (Voss et al., 2023). While the tables store the clinical data and related metadata of interest, the vocabularies provide *coded concepts* that precisely identify the type of measures, conditions, procedures, and other clinical events described in table records.

**FIGURE 3:**
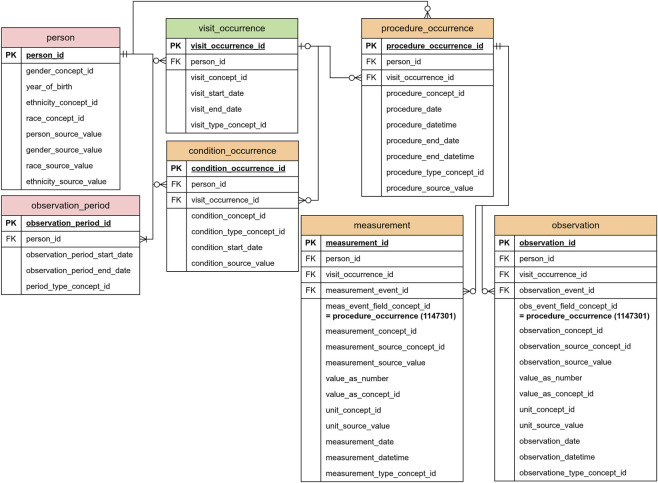
The proposed data model structure. Instances of the red tables are required for each patient. Orange tables store information about the ECG signals, as well as the extracted annotations, arrhythmias, patient diagnoses, and HRV indices. The main foreign keys and relationships required to associate records of different tables are shown. The *visit_occurrence* table acts as a connector between ECG procedures and conditions revealed by ECG signals.

The first step is to design the data model structure (Figure 3), for which we followed the official OMOP CDM v5.4 specifications (https://ohdsi.github.io/CommonDataModel/cdm54.html). The patient-centric architecture of OMOP CDM demands the person table to be always populated, connecting records (e.g., clinical conditions, observations, and measurements) related to a specific patient and facilitating longitudinal studies. Additionally, at least one observation_period instance is needed for each patient, representing time intervals during which procedures and visits are performed. The other tables required for the proposed schema are procedure_occurrence, condition_occurrence, visit_occurrence, measurement, and observation. Specifically, the first stores details about the performed examinations, such as the ECG acquisition type (e.g., “Ambulatory ECG” or “24 Hour ECG”) and date. We employ condition_occurrence to describe ascertained patient diagnoses and abnormalities reported in the annotation files or automatically detected from raw signals through the selected DNN. The visit_occurrence table is used to link the diagnoses documented in the previous table to the ECG procedures during which they were detected. Finally, records of the measurement and observation tables store primary ECG signal metadata (duration and sampling rate) and the calculated HRV indices, which are linked to their corresponding ECG procedures through dedicated attributes. Our usage of each table and the information we map to each attribute is described in detail in the following subsections.

#### 2.3.1 The *person* table

In the data schema designed for our application, in addition to the required attributes (person_id, gender_concept_id, year_of_birth, race_concept_id, and ethnicity_concept_id), the person table holds the source_value of person, gender, race, and ethnicity to ensure completeness of the reported information. In particular, patient_source_value stores the pseudonymized code assigned to the patient in the original dataset, enabling recognition and correct linkage if new data for the same patient are added in the future. Concerning the ethnicity attribute, reporting the “unknown” source value was necessary as none of the PhysioNet datasets we considered included this information, and the closest concept we could find among the valid ones for the ethnicity_concept_id field (which must be selected from standard *Ethnicity* domain concepts) was *1546846-More than one ethnicity*.

As instructed by OMOP CDM specifications, we selected the code *0-No matching concept* to populate the race_concept_id field for all person records, since none of the considered PhysioNet datasets reported this information. Notably, patient ethnicity and race, as defined by OMOP, are also expected to be missing in research datasets collected from the Italian hospitals involved in the HBD project, making the public datasets we selected a valid test bench for this specific aspect. Eventually, the year_of_birth field was populated by subtracting the patient’s age from the year in which the ECG data were acquired.

#### 2.3.2 The *observation_period* table

Regarding the observation_period table, all the available fields are required by OMOP CDM specifications and were filled in accordingly. Since, in the PhysioNet datasets we took into account, all ECG records for the same patient were acquired close to each other, we decided to define a single record of observation_period for each patient. In particular, we defined observation_period_start_date and its corresponding end_date by identifying the first and last ECG acquisition dates for each patient. This design choice could be refined with real-world data by defining a maximum number of days between consecutive ECG procedures (or other clinical events) to motivate their inclusion within the same observation period. If one of such events happens too distantly from the previous ones, this should mark the beginning of a new observation period to be recorded in the corresponding table.

The concept to populate the period_type_concept_id field was selected as the closest applicable to our case, belonging to the *Type concept* domain of OMOP standardized vocabularies. For this field, we selected *32827-EHR encounter record* for all observation periods, as we do not have particular reasons to distinguish across different types of observation periods with the data examined in this work. With real-world data, this field can be used to keep track of the source from which data belonging to a specific observation period are retrieved.

#### 2.3.3 The *visit_occurrence* table

For each observation period defined in the previous table, we created a separate record of the visit_occurrence table (i.e., one *visit*) for every date in which one or more ECG data collection sessions were performed for an individual patient. In fact, we can expect multiple ECG procedures to be performed on the same day for a single patient. As these are separated through distinct records of the procedure_occurrence table in our schema, as we will show, and a single visit event is recommended to last at least 1 day in OMOP CDM specifications, this motivates the usage of each visit record as a container for all the ECG procedures performed on the same date for a single patient. The beginning and end dates of the visit, which in our case study always coincide, yet in real-world data may span more than 1 day during hospitalizations, are specified by the visit_start_date and visit_end_date attributes, respectively.

For the visit_occurrence table, we fulfilled the descriptive requirements of the examined ECG data by populating only the required attributes. In particular, the visit_concept_id field is populated with a standard concept that represents the type of visit performed. In our use case, coded concepts *9202-Outpatient Visit* and *32036-Laboratory Visit* were both applicable, as these visits’ duration is expected to be no more than 1 day, yet we selected the latter to stress the availability of records in the measurement table associated with the ECG procedure performed during the visit. With real-world ECG data, different concepts might be used, for instance, to distinguish ECG acquisitions conducted in an emergency situation (*9203-Emergency Room Visit*) or during hospitalization (*9201-Inpatient Visit*) from ordinary ones. Finally, the field visit_type_concept_id was populated with the concept *32827-EHR encounter record*, consistently with the one selected for the corresponding attribute of the observation_period table.

#### 2.3.4 The *procedure_occurrence* table

As briefly anticipated above, a separate record for each ECG acquisition is defined in the procedure_occurrence table, which stores some of the main properties of the collected signals. The required attributes person_id and procedure_concept_id were used to store, respectively, the foreign key linking to the patient and the type of ECG examination performed. Specifically, we selected the more general concept *4065278-Ambulatory ECG* to mark short ECG acquisitions (less than 30 min, in our specific implementation) where the 12 standard ECG leads were not all available, *4145308–12 Lead ECG* to indicate diagnostic data collections featuring all standard ECG leads, and *4098508–24 Hour ECG* to describe longer ECG recordings. As the OMOP CDM only allows for one procedure_concept_id to be specified for each procedure, these concepts were selected by considering the most salient a researcher might look for when searching for useful ECG examinations for a study.

Initially, we considered using the device_exposure table instead of procedure_occurrence to allocate basic ECG exam metadata. However, since the above concepts describing different ECG modalities belong to the *Procedure* domain, the latter table was selected.

The required attribute procedure_date is populated with the date on which the ECG examination was performed. This temporal information is further refined, for our use case, by populating the optional procedure_datetime, procedure_end_date, and procedure_end_datetime fields with the appropriate dates and times. In PhysioNet datasets, details on the beginning and end times of each ECG acquisition are rarely provided. Thus, we reconstructed this information by treating the data file creation date and time as the start of the ECG acquisition and adding the total duration of the recorded signals to estimate the end time of the ECG recording. In real-world data, the ECG acquisition date and time should always be provided in the header files complementing the ECG data, as this information ensures each ECG recording can be unambiguously linked to a specific visit.

Eventually, we use the optional attributes visit_occurrence_id as a foreign key to link each ECG procedure to the visit in which the ECG was collected and procedure_source_value to keep track of the path where the relevant ECG data files are stored. Although the limited length of the char array expected by this field in the OMOP CDM (VARCHAR(50)) may prevent saving long file paths, this heavily depends on the specific storage type adopted in the underlying infrastructure. For example, a character array of this kind would be sufficient to store the global unique identifiers typically used for object storage. Nevertheless, if longer character sequences are needed, an OMOP CDM note table can be added to store text of arbitrary length. Records of this table can be directly linked to procedure_occurrence entries by leveraging the note_event_field_concept_id and note_event_id fields, as will be explained in Section 2.3.6 for measurement and observation records.

#### 2.3.5 The *condition_occurrence* table

The condition_occurrence table stores all ascertained patient diagnoses and the abnormalities identified on the ECG signals, either those tracked in PhysioNet annotation files or those detected by the selected DNN. Depending on the method used to obtain each diagnosis, a different condition_type_concept_id is used to allow users of the data catalog to distinguish among the three types. For example, some researchers might need to analyze all ECG signals collected from patients with a confirmed diagnosis, whereas others might look for signals that exhibit signs of a certain arrhythmia, such as tachycardia, regardless of whether the condition has been confirmed by clinicians.

In particular, we selected *32840-EHR problem list* from the *Type concept* domain to identify ascertained patient diagnoses, found in PhysioNet header files; such a use of this concept is in line with the official recommendations, explicitly stating its appropriateness for “confirmed patient conditions” (https://github.com/OHDSI/Vocabulary-v5.0/wiki/Vocab.-TYPE_CONCEPT). We used *32827-EHR encounter record* to mark abnormalities found on the ECG annotation files and reviewed by expert clinicians; the use of the same *Type concept* adopted for the observation_period and visit_occurrence tables underlines that this condition was found during the visit. Finally, we attributed the *Type concept 32880-Standard algorithm* to the conditions found by analyzing the ECG signals through the selected DNN, implying that these were obtained automatically and may require further checks.

The specific condition_concept_id we selected to map every abnormality found in the considered PhysioNet datasets is reported in Tables 1, 3, 4, grouped in the three described condition types. As these tables show, all concepts were found in the *Condition* domain of the OMOP vocabularies, confirming the appropriateness of representing also automatically-detected conduction abnormalities in the condition_occurrence table. Indeed, OMOP CDM specifications recommend concepts belonging to the *Condition* domain to be represented only within this table.

The other required attributes of this table are person_id and condition_start_date, with the latter storing the date of the visit during which the patient’s condition was identified. Optional fields adopted in our schema are visit_occurrence_id, explicitly associating each condition record with the relevant visit, and condition_source_value, used to store the original label of the condition found in the ECG datasets.

#### 2.3.6 The *measurement* and *observation* tables

The measurement and observation tables can both be used to store numeric or categorical results and facts from medical examinations. According to OMOP CDM specifications, which of these two tables should be used to accommodate a specific result depends on the domain the concept_id selected to define the measure belongs to. If it is the *Measurement* domain, the value must be recorded in the measurement table; if it is others, the result must go in the observation table. *Condition*, *Procedure*, *Drug*, or *Device* are the only domains not permitted for concepts in the latter table. For this reason, we had to store the automatically detected ECG arrhythmias in the condition_occurrence table and could not represent them as observations made from ECG signals.

Apart from the rules on the concept_id field, the other attributes available for the two tables share similar definitions and content. In particular, we populated the foreign keys person_id, visit_occurrence_id, and measurement_event_id (or equivalent for the observation table). Particularly, the last field enables linking the measure to a record stored in any other table, allowing us to connect each measure (or observation) to the specific ECG procedure from which it was derived, by setting the meas/obs_event_field_concept_id attribute to the concept identifying the procedure_occurrence table (1147301) in the *Metadata* domain.

Regarding the other attributes included in our schema, we defined measurement_datetime as the beginning of the ECG procedure linked to the measure. The measurement_type_concept_id field was set to *32827-EHR encounter record* for every measure, in accordance with the *Type concept* chosen for the related ECG procedure. The appropriate unit_concept_id was assigned to measures for which units of measurement were encoded through standard concepts in the OMOP vocabularies; when these were not available, we relied on the unit_source_value field to store this information. Finally, the value_as_number attribute allocates the numeric result of the individual measure, whereas value_as_concept_id was included to store possible categorical results from future pipeline versions.

The corresponding fields of the observation table were used for those measures for which fully descriptive concepts belonging to the *Measurement* domain could not be identified. In particular, as detailed in Table 2, all the calculated HRV metrics were stored in the measurement table, as well as the duration of the recorded ECG signals, whereas the sampling frequency was the only parameter requiring insertion in the observation table.

#### 2.3.7 Identification of concepts and custom *vocabulary*, *concept*, and *concept_relationship* entries

After defining table structure and interconnections, appropriate concepts must be selected to describe the information (patient diagnoses, ECG-derived annotations, conduction abnormalities, and HRV indices) retrieved during the Extraction phase. A fundamental OMOP CDM rule, in this sense, states that the concepts used in every <tableName>_concept_id field must be marked as *standard* in the OMOP vocabulary for the specific domain required by each table.

The search for suitable standard concepts was performed with the aid of Usagi and Athena, open-source software tools designed to assist ETL developers in mapping codes from the source dictionary (i.e., the terminology used in the original datasets) to OMOP vocabulary standard concepts. Both tools allow for searching the vast collection of OMOP vocabularies, which comprises a number of established clinical ontologies, including SNOMED and LOINC, whose terms are interconnected via relationships that facilitate concept mapping across different ontologies. However, despite the remarkable completeness of terms offered by OMOP vocabularies, it is not always possible to identify standard concepts that accurately describe certain measures, observations, and other clinical entities.

In these situations, OMOP CDM specifications recommend identifying a semantically-close *standard* concept to associate with a well-descriptive *non-standard* concept. The selected *standard* concept should be mapped to the usual <tableName>_concept_id field, whereas an appropriate *non-standard* concept, if already available in the OMOP vocabularies, should be selected as a <tableName>_source_concept_id. By doing so, the description provided by the identified more general standard concept is augmented by the selected non-standard concept, in full compliance with the OMOP CDM, as both the concepts (i.e., the standard and the non-standard one) will be interpreted correctly by most OMOP analytic tools, including ATLAS, and be usable in queries. For example, in our application, the “ECG sampling rate” was precisely described by combining the standard concept *4117495-Sampling - action* from the *Observation* domain, expressed in the observation_concept_id field, with a perfectly matching non-standard concept, *37533243-Digital Sampling Rate*, stored in the observation_source_concept_id attribute.

On other occasions, we may be able to identify a sufficiently close standard concept in the OMOP vocabularies, as previously, but not a fully descriptive non-standard one. In this case, OMOP CDM specifications allow us to create the latter as a custom concept and link it to the identified standard one via a “Maps to” relationship (and a corresponding “Mapped from” in the opposite direction). Adding the specified relationships between the created *custom non-standard* concept and the identified standard one is crucial for the development of ETL routines that can automatically select the associated standard concept given the defined non-standard one. Furthermore, it allows custom concepts to be easily remapped to more appropriate standard concepts should they become available in future versions of the OMOP vocabularies. Updated details on this process can be found in a dedicated, recently-released section of the official documentation (https://ohdsi.github.io/CommonDataModel/customConcepts.html).

Eventually, if a semantically-close standard concept cannot be found to populate the required <tableName>_concept_id field, the only remaining alternative is to create a *custom standard* concept, following a procedure similar to the above one but with the difference that the custom concept will be defined as *standard* and the “Maps to/from” relationship will point to the concept itself. In the developed ECG pipeline, most extracted HRV features required the definition of custom standard concepts to be properly represented in the OMOP CDM. Specifically, we defined a custom record for the vocabulary table and populated the required fields, following the instructions provided at the above link. Then, we created one new record in the concept table for each measurement missing a precise standard concept in the OMOP vocabularies, linked each concept to the previously created vocabulary (through the vocabulary_id field), and assigned each a concept_id greater than 2 billion to distinguish custom concepts from those available in the OMOP vocabularies. We defined appropriate concept_name and concept_code to accurately describe the added concepts, assigned them to the *Measurement* domain (through the domain_id attribute), and declared them as standard by setting the “S” flag in the standard_concept field. Finally, in the concept_relationship table, we established both the “Maps to” and “Maps from” relationships from the new custom concept to itself, as required for standard concepts in the OMOP vocabularies to ensure consistency.

It should be noted that relying on custom vocabularies poses severe limitations to the usability of custom concepts outside the federation in which the vocabulary was developed. Particularly, it reduces the semantic interoperability of the database in network studies. Therefore, if a feature is not judged sufficiently clinically or research-relevant to justify defining a custom standard concept, it may be discarded to maintain the CDM fully specified within the available OMOP vocabularies. These assessments should be conducted by working groups of experts identified within the federation for each knowledge domain, with modalities that will be clarified in the Discussion.

**FIGURE 4:**
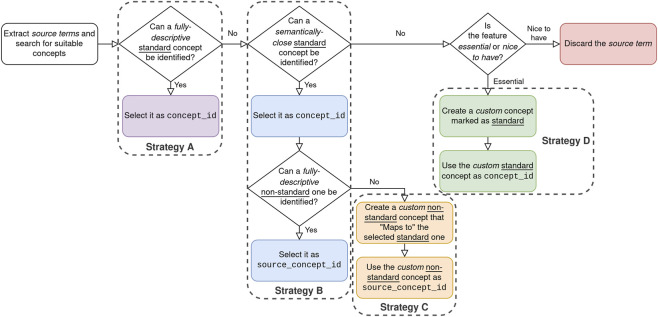
The adopted workflow for mapping source terms to OMOP vocabularies or custom concepts, as applied during the Transform phase of the ETL pipeline.

Figure 4 summarizes the mapping strategy detailed above, which we followed to map all the features that were extracted from the considered ECG datasets.

### 2.4 Implementation of transform and load steps

In the proposed pipeline, the functions MAINtransform and MAINload orchestrate, respectively, the transformation of the extracted features according to the described schema and the loading of the resulting tables into an OMOP CDM database. Specifically, transformation is performed by invoking a set of functions, each one dedicated to building a single OMOP CDM table or a set of interconnected ones. The separation into multiple functions facilitates the future inclusion of additional features or tables in the mapping procedure, as well as changes to how current ECG metadata are transformed into the various OMOP CDM tables.

These tables, as returned by MAINtransform, are then loaded into the OMOP CDM database, after checking for the existence of the same records in the database. MAINload performs both this check, primarily based on comparing the <tableName>_source_value fields of records present in the database with those to be loaded, and the insertion of new records. To use the function, a PostgreSQL database should have already been configured with the OMOP CDM v5.4 schema and the standard vocabularies imported.

## 3 Results

This section presents results from the implementation and testing of the proposed ECG data integration pipeline on the three public datasets considered for its development (Sections 3.1 and 3.2) and on the PTB-XL database, introduced for performance evaluation (Section 3.3).

Specifically, Section 3.1 shows the outcomes of the concept mapping process, which was conducted by applying the reasoning schema summarized in Figure 4 to the extracted conduction abnormalities, annotations, diagnoses, and HRV indices. In Section 3.2, we show the practical utility of the implemented data schema through example queries, illustrating how concept-based representations enable flexible retrieval and analysis of ECG data. Finally, Section 3.3 assesses the scalability of the pipeline, reporting its application to a large-scale dataset and providing empirical measures of computational efficiency across the ETL stages.

### 3.1 Concept mapping

The concepts selected to map patient diagnoses, conduction abnormalities, detected arrhythmias, extracted HRV features, and ECG signal properties are reported in Tables 1–4, along with the mapping approach adopted.

Specifically, the *Source term* table column shows the definition of each feature, as provided in the original datasets. *Map strategy* indicates the approach followed to map each source term to the matching concept identified in the OMOP vocabularies: *Standard* refers to the approach presented as “Strategy A″ in Figure 4; *Standard plus Non-standard* refers to “Strategy B”; *Custom Standard* indicates the approach marked as “Strategy D″, whereas *Custom Non-standard* (not needed in our mapping, hence not appearing in the tables) indicates “Strategy C”. The *Concept ID* column provides the code of the selected semantically-matching concept in OMOP vocabularies (≤2,000,000,000) or added custom vocabulary (>2,000,000,000) . *Concept name* shows the definition of the selected concept (i.e., Concept ID), as reported in the vocabulary. Eventually, *Domain* reports the specific OMOP domain to which each selected concept belongs, which is crucial to understand some mapping choices explained in Section 2.3.

In summary, all 7 patient diagnoses, 16 pathological annotations from PhysioNet ECG annotations, and 6 automatically detected conduction abnormalities could be accurately mapped relying on standard concepts from OMOP vocabularies, in complete adherence to OMOP CDM requirements. Of the concepts selected for these categories of features, only a few were used multiple times to define different source terms in Physionet ECG annotations with similar meanings (e.g., 4327066 denotes both ventricular and supraventricular escape beats), underscoring the precision with which most conditions could be represented as distinct entities. For the evaluated signal properties, signal duration was mapped to a standard concept, whereas signal sampling rate required combining a more general standard concept with a perfectly matching non-standard one. On the other hand, of the 15 HRV indices evaluated, only 2 (AVRR and SDRR) could be mapped to standard concepts already available in OMOP vocabularies. The remaining 13 HRV indices required the addition of a custom vocabulary and *custom standard* concepts, as no semantically-close standard or non-standard concepts were available in the OMOP vocabularies to describe them properly, making “Strategy D” (Figure 4) the only viable option for these features.

This result reflects the richness of diagnostic, procedure-, and patient-related information characterizing OMOP vocabularies, yet suggests a shortcoming in their current ability to represent outputs from advanced health data processing. Indeed, incorporating terms from custom vocabularies helps mitigate this problem by enabling accurate descriptions of measures, observations, and conditions that have not yet been defined in the shared OMOP vocabularies. However, this solution applies only to local or federated OMOP implementations, in which the custom vocabulary can be easily shared with other federation members. In fact, given the complexities that would arise from sharing custom vocabularies in network studies (Abellan et al., 2025), exploiting custom concepts reduces data reusability. This is especially true for *custom standard* concepts, given that they cannot be mapped to any other concepts already available in the OMOP vocabularies, as explained in Section 2.3.7. As a result, features identified through *custom standard* concepts would not be available in network studies. Interestingly, this limitation does not apply to features mapped using a non-standard (even *custom*) concept in the source_concept_id field, such as the ECG sampling rate, as the more general standard concept to which the non-standard one “maps to” would be available, instead, and made accessible by OMOP-compliant software tools in place of the non-standard one.

### 3.2 Examples of significant queries

To demonstrate the advantages and highlight current limitations of the proposed pipeline, this section presents results from queries executed on the OMOP CDM database after loading the tables created as illustrated in the previous sections. The exact queries used to retrieve these results are provided in the pipeline repository, linked at the beginning of Section 2.

A major advantage of mapping features, observations, and conditions to standard concepts (either provided by OMOP vocabularies or custom ones) is that they enable analyzing the specific entity they recall by referring to the same concept_id, independently of the original source of information. Thus, by referring to these unique concepts, researchers can filter ECG records based on specific annotations or measurements to select relevant data for the analysis of interest. In particular, this may facilitate the retrieval of more relevant records by simultaneously querying multiple modalities that return the same type of information. Thanks to the use of concepts, researchers do not need to know the source term with which each modality returns the specific information they look for, as it will be shown in the following.

Let’s consider the concepts in common between the set of abnormalities provided by PhysioNet standard ECG annotations (Table 1) and those detected by the selected DNN (Table 3). These modalities can both inform about the presence of a left or right bundle branch block condition. However, the first method returns these conditions with the source terms “Left bundle branch block” and “Right bundle branch block”, while the second uses the labels “LBBB” and “RBBB” to match the same diagnoses. To retrieve all ECG records exhibiting these specific conditions, without distinguishing between the two modalities, we can easily refer to the concepts identifying the two conditions (LBBB: 316998, RBBB: 314059), without requiring knowledge of all the associated source terms. A query performed with this approach returns the result observed in Table 5. In this query, the COUNT operator was applied to the returned ECG procedures to aggregate the results; however, the related raw ECG signals that the user might wish to analyze can be easily retrieved by referring to the procedure_source_value field. It is worth noting that a search based solely on the standard annotations provided by PhysioNet (stored with the type concept: “EHR encounter record”) would have yielded a small number of ECG records (i.e., four), underscoring the advantage of including an automatic abnormality-detection method in the feature-extraction step of the pipeline.

**TABLE 5:** Counts of ECG procedures presenting the conditions LBBB or RBBB.

No. of ECG records	Concept ID	Source term	Type concept name
27	314059	RBBB	Standard algorithm
4	314059	Right bundle branch block beat	EHR encounter record
24	316998	LBBB	Standard algorithm

Standardized concept IDs (*316998-LBBB* and *314059-RBBB*) enable retrieving all ECG signals exhibiting RBBB or LBBB regardless of the source term and the modality with which the information was obtained (i.e., ECG annotations or automatically-detected conduction abnormalities).

Another advantage of the proposed approach is that it enables rapid exploratory analyses of the database, even combining information from multiple types of extracted features. For example, we can perform a query to return all the ECG procedures for which the selected DNN detected ST or BT arrhythmias and examine the average AVRR measured in these records, grouped by the type of arrhythmia. As Table 6 shows, the decrease in AVRR expected during ST and the increase during BT is already noticeable if we analyze all types of ECG procedures (i.e., longer and shorter recordings) together. However, when we group the results by ECG acquisition type (Table 7), we observe that shorter recordings (12 lead ECG: 4145308) show a more pronounced difference between the two conditions than longer ones (24 hour ECG: 4098508). This is because the current version of our pipeline estimates HRV indices from the entire ECG traces, making the evaluation of AVRR less specific to the detected SB or BT episodes in longer signals than estimates obtained from shorter recordings. A possible development to overcome this limitation is discussed in Section 4.1. Of course, the addition of the second GROUP BY instruction in the query, to separate results by recording type, reduces the number of samples providing support to our (trivial, in this case) research question, reminding researchers of the importance of including these considerations when evaluating the robustness of similar exploratory analyses.

**TABLE 6:** Average AVRR of ECG procedures presenting the automatically detected conditions SB or ST, which were selected relying on the corresponding concept IDs identified in the OMOP vocabularies.

No. of ECG records	Concept ID	Concept name	Average AVRR (ms)
38	4171683	Sinus bradycardia	1079
78	4007310	Sinus tachycardia	765

The average AVRR shows the expected difference between the two conditions, with SB having increased mean RRI duration compared to ST.

**TABLE 7:** Average AVRR of ECG procedures presenting the automatically detected conditions SB or ST, further grouped by procedure type.

No. of ECG records	Concept name (Procedure)	Concept ID (Condition)	Concept name (Condition)	Average AVRR (ms)
26	12 lead ECG	4007310	Sinus tachycardia	502
9	12 lead ECG	4171683	Sinus bradycardia	1286
52	24 hour ECG	4007310	Sinus tachycardia	897
29	24 hour ECG	4171683	Sinus bradycardia	1014

Grouping the ECG procedures also by procedure type (i.e., 12 lead ECG vs. 24 hour ECG) highlights the separation between SB and ST in terms of average AVRR, as compared to Table 6.

These and similar queries can also be used to double-check the appropriateness of the applied ETL routine for the processed datasets and to verify that the association between source terms and selected OMOP concepts was performed as expected. For example, SQL queries combining SELECT and COUNT can be used to check the number of patients and ECG exams added to the OMOP database at the end of the Load step. Likewise, queries relying on the SELECT DISTINCT operator after JOINing condition_occurrence and concept tables can be used to inspect the associations between source terms appearing in the original datasets and the corresponding concept names selected from the OMOP vocabularies. The last type of query can also be used to present clinicians with a unified view of this association and help them ultimately validate the concepts selected for mapping.

### 3.3 Pipeline scalability evaluation

The ability of the proposed ETL pipeline to scale to massive ECG datasets was tested on the publicly available PTB-XL dataset. Since high-resolution ECG signals (500Hz sampling rate) are also provided in the dataset, we selected these instead of the low-resolution ones (100Hz sampling rate) to analyze performances with data closer in format to those collected in clinical applications.

All 21799 ECG records were processed without issues. Given that every ECG signal lasts 10 s and features all 12 standard leads, the automated detection of conduction abnormalities through the selected DNN could be performed for all signals. The time needed to run the ETL pipeline on the entire dataset was 42.4 h, using a common workstation (see specs in Section 2.1). As expected, running the Extraction phase took most of this time (99.87% of the entire ETL, 7.0 s per ECG record), with the DNN prediction requiring 0.4 s for each signal (5.7%) and the ECG processing, RR interval extraction, and HRV metrics estimate accounting for the remaining time (6.6 s, 94.3%).

The Transform step required 172.7 s in total (0.11% of the ETL) to generate the expected OMOP CDM tables. Finally, the Loading step required only 34.0 s (0.02% of the ETL) to be completed and inserted into the PostgreSQL database the following main records.

- 18869 new records for the person table, one for each individual patient available in the dataset;
- 21799 records for the procedure_occurrence table, one for each ECG exam;
- 6513 records into the condition_occurrence table, one for each conduction abnormality detected by the adopted DNN;
- 43552 records into the measurement table (all “ECG Duration” and AVRR measures, as the other HRV metrics could not be estimated with 10-s long ECG signals);
- 21799 records into the observation table, one “ECG Sampling Rate” entry for each signal.

It is worth mentioning that the Extraction step’s processing time would be significantly reduced on multi-core systems by introducing parallelization through the Single Program Multiple Data (SPMD) paradigm. This extension, which we envision mainly for production environments, will be made available in a future version of the pipeline implementation.

## 4 Discussion

This work presented a standardized and modular pipeline for integrating heterogeneous ECG datasets into an OMOP CDM database, with the aim of improving interoperability, findability, and reuse of biosignal-derived information in multicentric research. Starting from publicly available ECG datasets with diverse acquisition characteristics, the pipeline harmonizes raw data and metadata through validated preprocessing routines, extracts clinically relevant annotations, conduction abnormalities, and HRV features, and structures them within the designed OMOP CDM schema. The extracted features were semantically mapped to the OMOP standard vocabularies, and the results of this process highlighted both the remarkable availability and specificity of concepts for diagnoses and annotations and the limitations encountered when representing measures from signal-processing outputs, such as HRV indices. Specifically, all patient diagnoses and ECG-derived conditions could be encoded using standard concepts, whereas most HRV measures required custom concepts, underscoring current gaps in OMOP vocabularies. Finally, example queries illustrated how the proposed representation enables flexible retrieval of ECG records across datasets and feature-extraction methods, supporting data discovery and exploratory analyses that combine signal characteristics, derived features, and clinical conditions.

One of the main challenges encountered in the mapping procedure was defining custom concepts to accurately represent all the HRV indices. This was required because only features identifiable by means of standard concepts can be queried and analyzed using software tools provided by the OMOP community. However, as anticipated in Section 3.1, the usability of custom vocabularies is constrained in network studies, deeply affecting the reusability of data by research centers outside the federation. To overcome this limitation and make it possible to use custom concepts in network studies, a request for their inclusion in the OMOP vocabularies can be submitted following a dedicated procedure (https://ohdsi.github.io/CommonDataModel/vocabRequest.html). Specifically, either the inclusion of existing ontologies featuring the concepts of interest or the addition of single custom concepts, if they are unavailable in any other ontology, can be requested. The former approach is recommended as it allows reusing knowledge and concepts that have already been defined in external vocabularies, typically after extensive discussion within a community of experts in a specific field.

Federations supported by expert clinical researchers should pursue the expansion of existing OMOP vocabularies and rely on custom vocabularies only as a temporary solution. In fact, although this path would require a non-negligible amount of time and resources to properly define new concepts or search external vocabularies for appropriate ones, it would also contribute to expanding OMOP’s semantic capabilities, benefiting the entire community. For example, a recent study proposed the integration of the OMOP vocabularies with concepts related to perinatal research (Abellan et al., 2025), demonstrating that these extensions are not only feasible but also welcome within the community.

The adoption of custom vocabularies, while it may be necessary to accurately represent features not yet covered by OMOP vocabularies, raises concerns regarding semantic fragmentation and reduced interoperability even across sites within the same federation. To mitigate these risks, proper management of custom vocabularies is required, particularly in governing the process by which new concepts can be proposed within the federation. Indeed, allowing all federation members to create their own custom concepts to define additional features that each may need in their pipelines would rapidly lead to a steep increase in the number of adopted custom concepts, ultimately undermining the purpose and principles on which OMOP CDM is founded. As a possible solution, we suggest the adoption of a governance model in which custom concepts are not introduced *ad hoc* by individual institutions, but are instead managed through a single federated vocabulary shared by all members of the research federation. In this context, the definition of minimum sets of data and metadata to be represented in the CDM - including derived ECG features - should be the outcome of coordinated decisions made at the federation level, supported by dedicated working groups of experienced clinical researchers. These groups are responsible for assessing the scientific relevance, robustness, and reproducibility of candidate features, and for agreeing on whether their inclusion justifies extending the federated vocabulary. Importantly, this process is inherently dynamic: as new evidence emerges, new features may become relevant, while others may be deprecated. To ensure transparency and traceability of these decisions, such governance mechanisms can be implemented through collaborative, Git-based workflows, where proposals for changes to the federated custom vocabulary are submitted, reviewed, and discussed via issue trackers and pull requests.

A similar careful approach should be adopted to balance completeness against long-term maintainability of the designed standardized integration pipeline. While an exhaustive mapping of many useful features may appear desirable, attempting to standardize measures whose clinical relevance, robustness, or extraction parameters are still under debate in the scientific community can substantially increase pipeline complexity without providing additional analytical value. For this reason, the feature selection operated through the definition of minimum data and metadata sets, as explained above, should also be directed to selecting features for which reliable, well-established extraction pipelines are available. Indeed, expanding the set of extracted features directly impacts maintainability by increasing the number of pipeline modules to be developed, maintained, and integrated with preexisting ones, as well as the complexity of the Transform phase, and the effort required to identify suitable semantic mappings. Moreover, including more features raises the likelihood of resorting to custom concepts, given the illustrated limitations of OMOP vocabularies when it comes to representing quantitative outputs from advanced raw data processing.

Finally, as the results reported in Section 3.2 show, a significant advantage of implementing a data standardization pipeline as the one proposed in this work is that it enables data querying using the same syntax independently of the source and modality with which the information was extracted. In fact, researchers can expect the same data fields and associated coded concepts to be available once the source has been transformed into the standardized OMOP dataset. This facilitates, for example, the selection of data of interest for a study and the retrieval of larger patient cohorts, significantly improving data discovery in large data-sharing or analytics platforms. It is also important to note the scalability of the concept-based record selection approach shown in the examples above. As additional methods for the automatic detection of, for instance, specific conduction abnormalities become available, the federation may choose to incorporate them into the pipeline. While different methods may identify the same abnormality under distinct source labels, the growing number of detection algorithms makes it increasingly important to represent these labels through encoded concepts in order to limit query complexity. This approach would enable researchers, for example, to retrieve ECG records identified by all available automatic detection algorithms collectively - thereby prioritizing quantity over quality - or, conversely, to intersect the results produced by multiple methods to increase the reliability of the detected diagnoses, effectively prioritizing quality over quantity.

Though selecting ECG records based on automatically detected abnormalities can be convenient, particularly for exploratory analyses, limiting the scope of the analysis to clinically confirmed diagnoses may be crucial to achieve reliable conclusions in some cases. With our mapping strategy, researchers can refine their search by filtering ECG records using the condition_type_concept_id field and retaining only those that match the *32840-EHR problem list* concept, representative of clinically confirmed diagnoses.

### 4.1 Current limitations and future developments

A current limitation of the proposed ECG standardization pipeline is that its development was based exclusively on publicly available ECG datasets, specifically on selected repositories from PhysioNet. This choice was motivated by the need to release a pipeline that could be readily tested, reproduced, and customized by the wider research community without data access constraints. PhysioNet datasets provide a favorable compromise between heterogeneity and structural coherence: while they share comparable file formats, they still exhibit substantial variability in ECG signal characteristics, channel labeling conventions, diagnostic annotations, and contextual metadata, which required addressing both syntactic and semantic heterogeneity challenges during the Extraction and Transform phases. Although the pipeline was developed using the datasets described in this work, its modular design should enable straightforward adaptation to other, similarly structured PhysioNet ECG datasets, with limited code adjustments.

For instance, processing the PTB-XL database for performance evaluation (after developing the pipeline based solely on the other three datasets) required only copying patient information–originally provided in a separate CSV file–into the *header files* (.hea), following the format expected in standard PhysioNet datasets. The second and final step required to load all information available in the dataset into our OMOP CDM schema would have been to map the diagnoses extracted from the same CSV to suitable concepts identified within the OMOP vocabularies. However, mapping all the 71 SCP-ECG-formatted diagnoses represented in the dataset was deemed out of scope for the present work.

More in general, we expect ECG datasets different from PhysioNet’s to need only minor adjustments to be properly handled through the proposed pipeline. In particular, changes may be required to the input data formats, as the current pipeline version expects input data to conform with PhysioNet specifications and include, for each ECG exam, a.dat file storing the ECG traces and a.hea file with the relevant clinical and technical metadata. In hospital datasets, instead, ECG recordings are provided with diverse data formats, such as within EDF or CSV files, requiring the development of either an *adapter* to convert the original data into.dat files before pipeline application or a compatibility layer to integrate the current pipeline with the new data format. The same goes for ECG exam metadata, often provided in structured textual or tabular formats, such as JSON, CSV, or XLSX, instead of PhysioNet.hea files.

Importantly, the primary goal of this work is not to deliver a universal, ready-to-use solution for processing many different ECG data formats, but rather to provide a transparent and extensible framework that clinical researchers and developers can tailor to their own biosignal data sources. In this respect, the integration of real-world ECG data from clinical institutions is a key next step and is already planned within the HBD project, where collaborative development across participating institutes will enable the pipeline to be tested on operational data and refined to address practical clinical research questions.

As shown in Section 3.2, a second limitation in the current version of the pipeline is that HRV indices and, in general, ECG features, are evaluated based on the entire signals, making their interpretation less specific as the signal duration increases. A possible solution to this issue might be estimating the HRV indices within a moving window, setting a size compatible with the minimum signal duration required for each HRV feature to be calculated robustly (Section 2.2). The specific measurement_datetime field of the measurement table might then be used to indicate which portion of the signal a specific HRV index is referred to, considering the starting date and time of the procedure as a reference for the beginning of the ECG signal. A similar strategy may also be applied to increase the granularity of labels returned by automatic abnormality detectors, such as the DNN considered here, to provide predictions for each segment of the analyzed signals rather than a single label per ECG procedure. While enhancing data completeness and creating additional analytical opportunities, this solution would increase the complexity of the Transform step and the number of records generated in the final database, particularly for 24-h ECG recordings. Thus, the decision about whether to change the Transform phase in this direction should also factor in the capabilities of the infrastructure hosting the application and the amount of expected ECG data to be stored, in addition to the actual need of researchers in the federation for this fine level of granularity in the final database.

### 4.2 Concluding remarks

We presented the whole process followed to develop a pipeline for standardizing ECG-related information extracted from heterogeneous datasets. The current version of the pipeline is available in a public repository to encourage further extension and customization to meet researchers’ needs.

Results show that the OMOP CDM proved an effective method to homogenize information structuring and feature representation of ECG data from several sources. The definition of a common structure makes data more accessible for multicentric studies, encouraging the sharing of health data across institutes. Indeed, the adoption of standard concepts, when available, and agreed-upon mapping procedures, even relying on custom vocabularies as necessary, ensures the extracted features are recognizable within the federation. Precise governance mechanisms are required to manage and regulate the extension of both the pipeline and the federated custom vocabulary to mitigate risks deriving from excessive complexity and semantic fragmentation. Furthermore, the possibility of extending the applicability of custom concepts to network studies through OHDSI-regulated procedures encourages the adoption of OMOP CDM for feature-extraction pipelines from raw health data.

Given that renowned international initiatives, such as the EHDS, are adopting OMOP CDM as one of the accepted standards for data sharing (Hussein et al., 2024), standardization efforts like the one presented in this study can help scale health data reuse at the European level. However, the current lack of specialized terms to describe outputs from advanced processing techniques in the standard vocabularies partially hinders this potential, prompting the extension of the OMOP vocabularies in this direction.

Prototype applications and case studies are currently under definition in the HBD project to validate the proposed ECG standardization pipeline on Italian research hospitals’ data and extend the presented workflow to other biosignals.

## Data Availability

The original contributions presented in the study are included in the article, further inquiries can be directed to the corresponding author.
